# Clinical signs and symptoms in a joint model of four disease activity parameters in juvenile dermatomyositis: a prospective, longitudinal, multicenter cohort study

**DOI:** 10.1186/s13075-018-1687-8

**Published:** 2018-08-15

**Authors:** E. H. Pieter van Dijkhuizen, Maria De Iorio, Lucy R. Wedderburn, Claire T. Deakin, on behalf of the JDRG, Kate Armon, Joe Ellis-Gage, Holly Roper, Vanja Briggs, Joanna Watts, Liza McCann, Ian Roberts, Eileen Baildam, Louise Hanna, Olivia Lloyd, Susan Wadeson, Phil Riley, Ann McGovern, Clive Ryder, Janis Scott, Beverley Thomas, Taunton Southwood, Eslam Al-Abadi, Sue Wyatt, Gillian Jackson, Tania Amin, Mark Wood, Vanessa VanRooyen, Deborah Burton, Joyce Davidson, Janet Gardner-Medwin, Neil Martin, Sue Ferguson, Liz Waxman, Michael Browne, Mark Friswell, Helen Foster, Alison Swift, Sharmila Jandial, Vicky Stevenson, Debbie Wade, Ethan Sen, Eve Smith, Lisa Qiao, Stuart Watson, Claire Duong, Helen Venning, Rangaraj Satyapal, Elizabeth Stretton, Mary Jordan, Ellen Mosley, Anna Frost, Lindsay Crate, Kishore Warrier, Stefanie Stafford, Lucy Wedderburn, Clarissa Pilkington, Nathan Hasson, Sue Maillard, Elizabeth Halkon, Virginia Brown, Audrey Juggins, Sally Smith, Sian Lunt, Elli Enayat, Hemlata Varsani, Laura Kassoumeri, Laura Beard, Katie Arnold, Yvonne Glackin, Stephanie Simou, Beverley Almeida, Kiran Nistala, Raquel Marques, Claire Deakin, Stefanie Dowle, Charalampia Papadopoulou, Shireena Yasin, Cerise Johnson-Moore, Emily Robinson, Kevin Murray, John Ioannou, Linda Suffield, Muthana Al-Obaidi, Helen Lee, Sam Leach, Helen Smith, Anne-Marie McMahon, Heather Chisem, Ruth Kingshott, Nick Wilkinson, Emma Inness, Eunice Kendall, David Mayers, Ruth Etherton, Danielle Miller, Kathryn Bailey, Jacqui Clinch, Natalie Fineman, Helen Pluess-Hall, Lindsay Vallance, Louise Akeroyd, Alice Leahy, Amy Collier, Rebecca Cutts, Emma Macleod, Hans De Graaf, Brian Davidson, Sarah Hartfree, Danny Pratt

**Affiliations:** 1 Pediatric Rheumatology, University Medical Center Utrecht Wilhelmina Children’s Hospital, Utrecht, The Netherlands; 2 Pediatric Rheumatology, IRCCS G. Gaslini, Genoa, Italy; 3 Paediatric Rheumatology, University College London GOS Institute of Child Health, 30 Guilford Street, London, WC1N 1EH UK; 4 Department of Statistical Science, University College London, London, UK; 5 Great Ormond Street Hospital for Children, London, UK; 6 NIHR GOSH Biomedical Research Centre (BRC), London, UK

**Keywords:** Juvenile dermatomyositis, Disease activity, Bayesian model, Longitudinal data, Clinical associations

## Abstract

**Background:**

It is currently impossible to predict the prognosis of patients with juvenile dermatomyositis (JDM). The aim of this study was to find clinical features most strongly associated with outcome variables in JDM as a first step towards tailor-made treatment.

**Methods:**

In a large, prospectively followed, multicenter cohort study of 340 patients with JDM, each contributing multiple visits, a Bayesian model of disease activity was developed, using the four continuous outcome variables creatine kinase (CK), childhood myositis assessment score (CMAS), manual muscle testing of 8 muscle groups (MMT8) and the physician’s global assessment of disease activity (PGA). Covariates were clinical signs and symptoms. Correlations among visits of the same patient were resolved by introducing subject-specific random effects.

**Results:**

Myalgia and dysphonia were associated with worse disease activity according to all outcome variables. Periorbital rash, rash on the trunk, rash over large joints, nail fold changes and facial swelling were associated with higher PGA. Notably, periorbital rash was also associated with higher CK and lower CMAS and nail fold changes with lower CMAS. Contractures were associated with lower CMAS and MMT8 and higher PGA. Patients with higher CMAS exhibited a higher MMT8 as well. PGA had the highest probability among the four outcome variables of being abnormal even if the other three outcome variables were normal.

**Conclusions:**

The signs and symptoms associated with disease activity could be used to stratify patients and adapt treatment plans to disease activity. The correlation between CMAS and MMT8 and the unique information captured by PGA implied that PGA should be maintained as an outcome variable, whereas CMAS and MMT8 might be simplified.

**Electronic supplementary material:**

The online version of this article (10.1186/s13075-018-1687-8) contains supplementary material, which is available to authorized users.

## Background

The childhood inflammatory idiopathic myopathies (IIM) are a group of heterogeneous disorders, characterized by chronic skeletal muscle inflammation. Of these, juvenile dermatomyositis (JDM) is the most common, though still a rare disease with an incidence of about 1.9–4.1 per 1,000,000 children [1]. Its hallmark is muscle inflammation characterized by proximal muscle weakness, in concert with skin involvement presenting itself typically as Gottron’s papules, heliotrope rash, malar rash or erythema overlying the extensor surfaces of the joints [2]. JDM potentially involves other internal organ systems as well, most notably the gastrointestinal and the respiratory tracts and patients with major organ involvement have a poor prognosis [2]. JDM is heterogeneous in nature, in terms of disease severity and various patterns of involvement of muscle, skin and internal organ systems. About 24–40% of patients experience a monocyclic course, whereas 50–60% have chronic disease activity [2]. The mortality rate is around 2–3% [2].

Given the severity and burden of the disease and possible long-term complications, adequate treatment is of utmost importance [2, 3]. A recent trial has shown that the combination of prednisone and methotrexate has the best potential to induce disease remission in new-onset JDM [4]. Ideally, treatment is tailored to the patient, in such a way that patients with high disease activity, at risk of developing serious sequelae of the disease, receive early and aggressive treatment, whereas those with less severe forms of the disease receive milder therapy. Previous studies have revealed some clinical factors associated with a worse prognosis [1, 5, 6]. However, in these reports disease activity was either taken dichotomously at a single point in time or was analyzed as time to remission.

The aim of the current study was to find clinical signs and symptoms associated with higher disease activity as measured by four widely used continuous outcome variables, assessed longitudinally in a large, multicenter cohort of patients with JDM. Such associations with high disease activity could be used in follow up studies to predict disease outcome in patients with JDM as a first step towards tailor-made treatment.

## Methods

Patients were retrieved from the ongoing UK Juvenile dermatomyositis cohort and biomarker study (JDCBS), which started recruitment of patients with JDM across the UK in 2000 [7]. Patients were enrolled at diagnosis or shortly thereafter and followed up approximately every 3 months for 2 years and subsequently at least annually. At each visit, data were collected on signs and symptoms of the disease, such as skin manifestations (e.g., periorbital rash, periungual rash, Gottron’s papules, nail fold changes, ulceration), muscular involvement (e.g., muscle weakness, dyspnoea, dysphonia, dysphagia) and symptoms of involvement of other organ systems (e.g., arthritis, chest pain, abdominal pain, hematuria, melena; see Additional file 1 for full list). Blood was drawn for routine laboratory testing. Furthermore, data were collected on treatment and disease activity according to four widely used continuous outcome variables, i.e., creatine kinase (CK), childhood myositis assessment scale (CMAS) [8], manual muscle testing of 8 muscle groups (MMT8) and the physician’s global assessment of disease activity (PGA). These outcomes were selected because they have been validated as a set of parameters able to classify patients with JDM as active or inactive [9]. Furthermore, they are widely used and readily available in routine daily care. However, rather than applying previously published criteria for inactive disease and dichotomizing patients as active or inactive, we modeled the four parameters in a continuous way, thus taking full advantage of the information they contain.

Ethical approval was obtained by the multicenter ethical review board covering all participating institutions. All participants provided written informed consent, or age-appropriate assent with parental consent. The study was performed according to the declaration of Helsinki and good clinical practice guidelines.

The UK JDM study enrolled patients with suspected or definite myositis with symptoms starting before the 16th birthday. At the time of analysis, data on 469 patients from 4122 visits were available. Of these, 413 patients contributing 3881 visits met the inclusion criteria of having probable or definite JDM according to the Bohan and Peter criteria [10, 11].

### Statistical analysis

The data were analyzed using a Bayesian approach. Details of the analysis have been described elsewhere [12]. Briefly, a mixed effect regression model was fitted, by specifying a joint model for the four clinical outcome variables. These outcome parameters were modeled as continuous variables. This approach allowed us to account for the correlation among disease activity measures and, therefore, better exploit the information contained in the variables, compared to an analysis that considers the four outcomes as independent and treats the responses as dichotomous. In the analysis, CK values were log-transformed so that the distribution of CK was closer to normal. On the other hand, CMAS, MMT8 and PGA were square root transformed, as they are non-negative variables potentially assuming value zero. More interestingly, these three variables showed an excess of the best possible clinical value for that parameter for visits of patients in disease remission and a long tail towards the pathological end of the scale. These distributional characteristics must be accounted for in the analysis to avoid bias in the estimates. As such we modeled CMAS, MMT8 and PGA using an approach similar to hurdle models [13]. This approach allowed the model to estimate many more visits of patients to be in disease remission than a standard linear regression would do, thus mirroring the observed distribution of the outcome parameters.

To make maximal use of the information contained in the dataset, all visits of all patients were analyzed simultaneously. Temporal correlation between multiple visits of the same patient was accounted for using a subject-specific random intercept and, in the case of CK level, a subject-specific random slope for the time since diagnosis, in a way similar to a mixed model in a repeated measurements analysis [14]. Furthermore, due to correlations between the four outcome parameters, the four subject-specific random intercepts (one for each outcome variable) were modeled by specifying a multivariate normal distribution as random effect distribution. The covariance matrix of this distribution was estimated from the data [15]. This allowed the assessment of the correlation among the outcome parameters (i.e., the correlation that measures whether one outcome parameter tended to increase if another parameter increased as well within the same individual over time). Missing values in the covariates were imputed in the Bayesian model, by specifying an appropriate model for them. We did not impute missing values for the history variables, since this would amount to imputing a participant’s recollection, the accuracy of which is doubtful. Visits with any unobserved history variables were therefore excluded from the analysis.

The aim of the model was to find all clinical features associated with disease activity. Therefore, all clinical signs and symptoms and treatment variables, and the time elapsed since diagnosis were eligible to be included as independent variables in the model. Variable pre-selection was performed by selecting 50% of the variables using univariate linear mixed models. All treatment covariates and the time elapsed since diagnosis were included in the model, regardless of their performance in the univariate analysis. Bayesian variable selection on the pre-selected covariates was performed [16]. This approach yields the sparsest set of predictors that is still able to estimate the outcome parameters accurately. A predictor variable would either be included for all four outcomes or excluded.

The goodness of fit of the model was assessed visually by checking the fit of the model for patients with more than 10 visits, by looking at 95% Bayesian credible intervals (CI) for the disease trajectory over time. Furthermore, the ability of the model to predict values above or below the validated cutoff points (CK ≤ 150, CMAS ≥ 48, MMT8 ≥ 78 and PGA ≤ 0.2) [9], was assessed by calculating the scaled Brier score. This score has a range from 0 to 100% and has an interpretation similar to Pearson’s *R*^2^ statistic [17]. The out-of-sample prediction ability of the model was tested by randomly selecting five fully observed patients and leaving them out while fitting the model. The predicted values for the four outcomes over time were then compared to the observed values. Statistical analysis was performed in R 3.2.2 (R foundation for statistical computing, Vienna, Austria) and JAGS, using the package rjags [18].

## Results

Due to exclusion of visits with missing data on history variables, 340 of 413 patients were included in the analysis. Of these, the majority (69.4%) was female, the median age at diagnosis was 7.4 years (1st–3rd quartile 4.5–10.5) and disease duration since the onset of the first symptoms was short (median 0.3 years, 1st–3rd quartile 0.2–0.6). Patients contributing more visits to the study were more likely to have at least one visit without missing values in the history variables and were therefore more likely to be included. Excluded patients due to missing data in the history variables appeared to have shorter duration of follow up in the study, longer period of time after diagnosis before enrollment and less active disease (Table 1). The proportion of missing data in the analyzed data set was 7.3%.

**Table 1:** Baseline table

Parameter	Included	Excluded
	*N* = 340	*N* = 73
Female, *n* (%)	236 (69.4)	54 (74.0)
Age at diagnosis, years	7.4 (4.5, 10.5)	7.3 (4.1, 11.1)
Disease duration at diagnosis, years	0.3 (0.2, 0.6)	0.3 (0.2, 1.0)
Time after diagnosis at enrollment, years	0.2 (0.1, 1.1)	2.3 (0.4, 5.4)
Duration of follow up, years	4.1 (1.6, 7.1)	1.2 (0.1, 2.6)
Disease activity at enrollment:		
CK, U/L	103 (64, 440)	98 (45, 256)
CMAS, points	41 (21, 50)	46 (37, 52)
MMT8, points	65 (45, 80)	80 (64, 80)
PGA, cm	3 (1.3, 6.0)	2.3 (0.5, 4.0)

Values are the median (1st quartile, 3rd quartile), except where indicated otherwise

*Abbreviations: CK* creatine kinase, *cm* centimeter, *CMAS* childhood myositis assessment scale, *MMT8* manual muscle testing of 8 muscle groups, *PGA* physician’s global assessment of disease activity, *U/L* units per liter

**Fig. 1:**
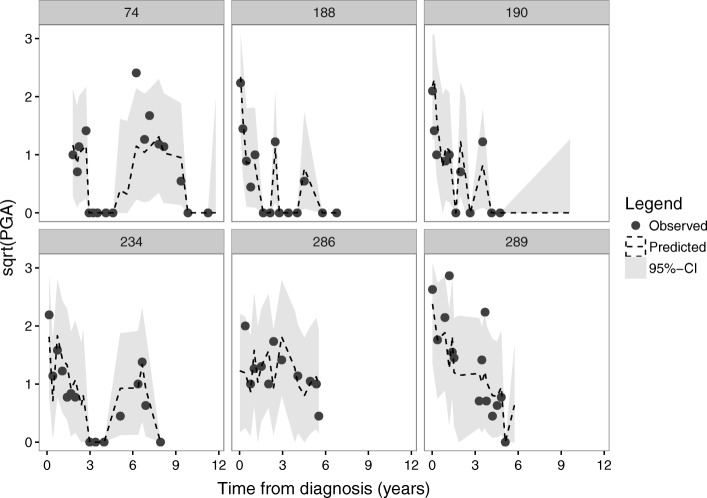
Goodness of fit of the physician’s global assessment of disease activity. Observed values of the (square root transformed) parameter (dark gray dots) for six randomly selected individuals were plotted against the predicted values by the model (dashed line) and the 95% CI (light gray area), showing that the predicted values corresponded well to observed patterns over time. Goodness of fit of the other outcome parameters was similar (not shown). Abbreviations: CI, credible interval; sqrt, square root; PGA, Physician’s global assessment of disease activity

The goodness of fit of the model was good as evidenced by a plot of the observed values versus the predicted values and 95% Bayesian CI (Fig. 1). Over all visits and outcome parameters, only 1.8% of observed values were outside of the CIs. The scaled Brier score was 49%, 42%, 63% and 80% of the maximally obtainable Brier score for CK, CMAS, MMT8 and PGA, respectively. The visual check of the out-of-sample predictions showed accurate predictions of the four outcomes. Only in two cases involving CMAS was the predicted value above the cutoff point for inactive disease (48 points), whereas the observed value was below, but in both these cases the observed value was close to the cutoff point (46 points and 47 points, respectively). The precision of the predictions was modest as evidenced by wide prediction CIs, owing to uncertainties in predictions, parameter estimation and missing value imputation.

**Fig. 2:**
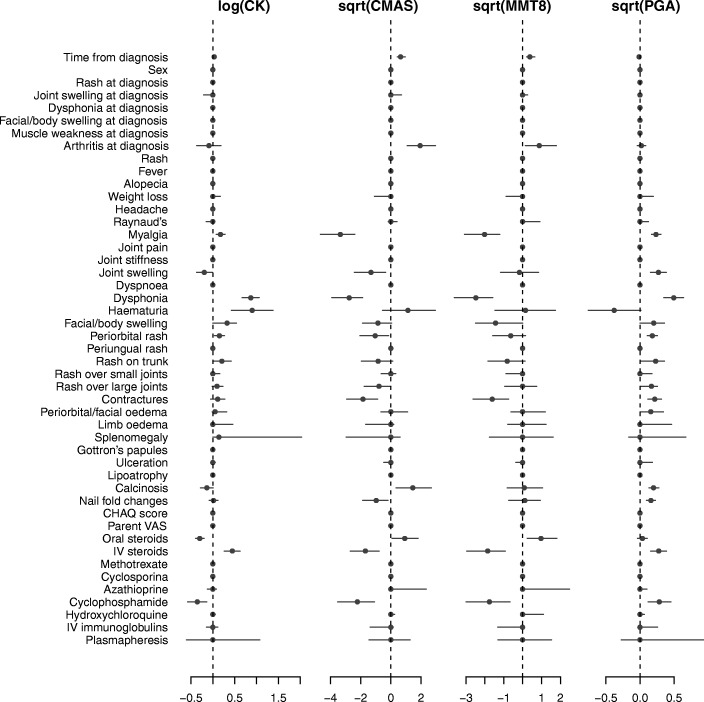
Regression coefficients and 95% credible intervals. Regression coefficients (dots) with 95% credible interval (horizontal lines) of the fixed effects of time elapsed since diagnosis and all covariates in the model, for all outcomes. Regression coefficients to the left of the vertical dashed line indicate the parameter is associated with lower values of the corresponding outcome measurement, and conversely for regression coefficients to the right of the dashed line. Some credible intervals are collapsed to 0 (e.g., association between sex and log creatine kinase (CK)), due to there not being any association at all. Abbreviations: sqrt, square root; CMAS, childhood myositis assessment scale; MMT8, manual muscle testing of 8 muscle groups; PGA, physician’s global assessment of disease activity

Estimates of regression coefficients of the continuous component of the model showed that CMAS, MMT8 and PGA tended to normalize over time, whereas hardly any influence of time on CK was noted (Fig. 2). Many muscular symptoms, such as myalgia and dysphonia were associated with higher disease activity. Cutaneous symptoms such as periorbital rash, rash on the trunk, rash over large joints, nail fold changes and facial swelling were associated with higher disease activity and, notably, were in some cases associated with more disease activity according to strictly muscular outcome parameters (e.g., periorbital rash was associated with higher CK values and lower CMAS and nail fold changes was associated with lower CMAS).

The presence of contractures was associated with lower CMAS and MMT8 values and higher PGA, whereas CK was not affected. An association was found between calcinosis and higher CMAS and PGA values. Joint swelling was associated with lower CMAS, lower CK and higher PGA and the presence of hematuria was accompanied by markedly elevated CK levels. Of all signs and symptoms at baseline, only arthritis was associated with increased CMAS and MMT8.

The use of both cyclophosphamide and intravenous (IV) steroids was associated with higher disease activity, though cyclophosphamide was also associated with lower CK levels. Conversely, oral steroids were associated with lower disease activity.

Correlations between the subject-specific random intercepts showed that patients with a higher CMAS tended to have a higher MMT8 as well (Table 2). Weak correlation was found between MMT8 and PGA and CMAS and PGA. All correlations between the subject-specific random intercept for CK and the other three parameters were low (Table 2).

**Table 2:** Estimated correlations with 95% credible intervals of subject random intercepts for the four outcomes CK, CMAS, MMT8 and PGA

Comparison	$\widehat{\rho}$	95% CI
CK vs. CMAS	0.07	(−0.07, 0.23)
CK vs. MMT8	−0.001	(−0.16, 0.15)
CK vs. PGA	0.0007	(−0.15, 0.16)
CMAS vs. MMT8	0.54	(0.42, 0.65)
CMAS vs. PGA	0.17	(0.01, 0.32)
MMT8 vs. PGA	0.23	(0.08, 0.32)

*Abbreviations: CI* credible interval, *CK* creatine kinase, *CMAS* childhood myositis assessment scale, *MMT8* manual muscle testing of 8 muscle groups, *PGA* physician’s global assessment

Of visits during which three outcome variables had normal values, the median (1st, 3rd quartile) probability that the predicted value for PGA remained abnormal (i.e. > 0.2) was estimated to be 52% (< 1, 78%). The median (1st, 3rd quartile) probability was 6% (2, 20%), 1% (< 1, 8%) and 1% (< 1, 17%), for CK, CMAS and MMT8 respectively.

## Discussion

This study identified clinical signs and symptoms associated with four outcome parameters taken continuously and longitudinally in a large, multicenter cohort of patients with JDM. This approach not only allowed to estimate the associations between various signs and symptoms and disease activity, but also enabled to assess correlations among the outcome parameters, in order to have a clearer understanding of disease activity by accounting jointly for all the outcome values. The results showed as expected that dysphonia, already known to be a marker of severe disease activity [2], was associated with higher disease activity. Hematuria was also associated with markedly elevated CK levels. Hematuria was measured by urine dipstick, which gives a positive result in the case of hematuria or myoglobinuria. In the former, hematuria is an indication of severe systemic (i.e., renal) disease. In the latter, myoglobinuria is due to severe muscular involvement, leading to rhabdomyolysis.

Cutaneous symptoms were associated with PGA, the only parameter taking account of cutaneous disease activity, but, interestingly, periorbital rash and nail fold changes were also associated with lower CMAS values and higher CK values, implying that cutaneous and muscular disease were correlated. It could therefore be hypothesized that the return of cutaneous symptoms in a patient in disease remission signals an imminent muscular relapse. This finding lends support to the expert-based opinion that ongoing skin disease reflects ongoing systemic disease activity [19]. Interestingly, Gottron’s papules, pathognomonic of JDM, were not associated with any outcome parameter.

The association between contractures and lower CMAS and MMT8 values might be due to difficulty in applying these instruments in the presence of contractures [20], especially given the absence of association with CK. Likewise, the association between calcinosis and higher CMAS levels might indicate that this phenomenon occurred in a late stage of disease and persisted in disease remission. Yet, the results showed that physicians gave higher PGA scores to patients with calcinosis.

Arthritis at diagnosis was associated with increased CMAS and MMT8, potentially due to a subset of patients with an overlap between JDM and juvenile idiopathic arthritis (JIA) with less severe muscular inflammation [2]. Joint swelling at the visit was associated with a higher PGA and lower CMAS, possibly indicating difficulty in executing the tasks of the CMAS in the presence of arthritis.

The substantial correlation between the subject-specific random intercepts for CMAS and MMT8 indicated that patients with a higher CMAS tended to have a higher MMT8, as reported previously [20]. This implied that information captured by CMAS was also partially captured by MMT8 and vice versa. Conversely, in many visits where CK, CMAS and MMT8 were normal, the model estimated PGA to be abnormal, implying that PGA captures aspects of disease that are not measured by the other outcome parameters. This was consistent with the observation that PGA was the only outcome parameter taking account of cutaneous involvement and involvement of other organs [21, 22]. Similar estimates for the other outcome parameters in our model were much lower, indicating that these variables captured information already conveyed by the other outcome measures. In conclusion, our observations supported the previously made proposal to increase the weight of PGA in the evaluation of JDM disease activity, to account for cutaneous disease activity [22]. Moreover, our results suggested that the three muscular disease activity measures, mainly CMAS and MMT8, could be shortened or even summarized into a single instrument, thus saving precious time during busy clinics, whilst retaining the same level of information.

The results of our study were in line with results obtained previously [1, 5, 6]. In a large cohort of 490 patients with JDM, analyzing the dichotomized outcome parameter at one time point, a mean of 7.7 years after diagnosis, there was association between CMAS and dysphagia and dysphonia [1]. This study also found an increased probability of having a CMAS score of 52 points in patients with cutaneous symptoms at onset, whereas in our study cutaneous manifestations (though not at onset) were associated with a lower CMAS score [1]. In a Canadian cohort of 84 patients, the persistence of skin rash, especially Gottron’s papules, 3 months after diagnosis was associated with a longer time to remission [5]. However, given that disease remission was defined in this study as absence of skin rash (including Gottron’s papules), myositis and arthritis, this finding might be tautological [5]. Gottron’s papules were not associated with any of the four outcome parameters in our study. In a retrospective study of 61 patients with JDM, a lower skin disease activity score (DAS) at baseline was also associated with a monocyclic disease course [6].

A limitation to the current study is the lack of auto-antibody and muscle biopsy data, and indeed any other biomarkers in our data set. The antibody patterns in the childhood IIM have been studied extensively and were found to determine different subsets of the disease, characterized among others by the distribution of skin rash, contractures, dysphonia, dysphagia, and the outcome of the disease [23, 24]. It would be interesting to know if these different subsets entail varying prognoses. Furthermore, prediction of outcome could be aided by muscle biopsy findings [25, 26] and yet other biomarkers may be found among blood plasma cytokines, chemokines and other inflammation-related compounds.

The goal of our study was to find clinical signs and symptoms associated with four frequently used disease activity parameters [9, 22]. As a consequence, no other outcome parameters, mainly cutaneous disease activity measurements, were considered. Future work may address this limitation; however, this would entail cutaneous symptoms that form part of the outcome measurement no longer being available as predictors. Likewise, our study did not contain patient-reported outcomes, nor other important outcomes, such as physical function, damage or health-related quality of life.

Hardly any features at baseline were associated with disease activity at follow up. This was most probably because the model included also signs and symptoms during follow up, the associations of which overwhelmed associations with baseline variables. Future work may investigate the predictive ability of these signs and symptoms in the assessment of disease activity. Attention should also be paid to the predictive ability of CK, CMAS, MMT8 and PGA at baseline and efforts should be undertaken to find out what variables are key to predicting disease outcome in JDM in order to identify a parsimonious set of data that should be collected at all clinical visits.

Finally, the exclusion of visits with missing history variables, led to the exclusion of 73 patients who appeared to have lower disease activity. This was probably due to the fact they were enrolled in the study at a later time point after diagnosis. Moreover, their follow up was shorter than in patients who were included in the analysis (Table 1). Therefore, the exclusion of this biased subset of patients was not considered problematic.

## Conclusions

The associations between clinical signs and symptoms and four continuous disease activity measurements in this large, multicenter cohort of patients with JDM, who were followed longitudinally, open up possibilities to personalize treatment plans in JDM, by offering more aggressive treatment to patients with signs and symptoms associated with higher disease activity. Follow up studies may attempt to predict future disease activity using the clinical signs and symptoms that were associated with higher disease activity found in the current study. Furthermore, the associations highlight interesting patterns, such as the association between skin disease and muscular outcome measures. Finally, the correlations between the four outcome measurements provide insight into unique information captured by each parameter and might be helpful in the determination of a parsimonious set of outcome parameters in JDM, for example by summarizing the CMAS and MMT8 in a single instrument and increasing the importance of PGA in the evaluation of disease remission.

## Additional file

Additional file 1: List of available covariates. (DOCX 14 kb)

## Abbreviations

CI: Credible interval
CK: Creatine kinase
CMAS: Childhood myositis assessment scale
DAS: Disease activity scale
IIM: Idiopathic inflammatory myopathies
JDM: Juvenile dermatomyositis
JIA: Juvenile idiopathic arthritis
MMT8: Manual muscle testing of 8 muscle groups
PGA: Physician’s global assessment of disease activity
