# Supplementary material for: Clinical signs and symptoms in a joint model of four disease activity parameters in juvenile dermatomyositis: a prospective, longitudinal, multicenter cohort study
Source: Arthritis Res Ther. 2018 Aug 15;20:180. doi: 10.1186/s13075-018-1687-8 (PMC6094880; doi:10.1186/s13075-018-1687-8)
Supplement: Supplementary file 1 — List of available covariates. (DOCX 14 kb) [file 13075_2018_1687_MOESM1_ESM.docx]

**Additional file 1**

**List of available covariate data**

*Demographics*

Sex, age at diagnosis.

*History at diagnosis*

Rash, weakness, myalgia, dyspnoea, dysphonia, dysphagia, joint pain, joint stiffness, joint swelling, fever, alopecia, weight loss, fatigue, mouth ulcers, headache, irritability, chest pain, abdominal pain, diarrhoea, melena, haematuria, facial or body swelling.

*Physical examination at diagnosis*

Weakness, arthritis, Gottron’s papules, ulceration, oedema.

*Symptoms at visit*

Rash, weakness, myalgia, dyspnoea, dysphonia, dysphagia, joint pain, joint stiffness, joint swelling, Raynaud’s phenomenon, fever, alopecia, weight loss, fatigue, mouth ulcers, headache, irritability, chest pain, abdominal pain, diarrhoea, melena, haematuria, facial or body swelling.

*Physical examination at visit*

Periorbital rash, periungual rash, rash on trunk, rash over small joints, rash over large joints, Gottron’s papules, nail fold changes, ulceration, calcinosis, lipoatrophy, arthritis, contractures, oedema, periorbital or facial oedema, limb oedema, trunk oedema, hepatomegaly, splenomegaly.

*Measurements at visit*

Childhood health assessment questionnaire, parent global assessment of well-being, parent assessment of pain.

*Treatment*

Methotrexate, oral steroids, intravenous (IV) steroids, cyclosporine, azathioprine, cyclophosphamide, hydroxychloroquine, IV immunoglobulins, plasmapheresis.
